# The Utilization of Brillouin Microscopy in Corneal Diagnostics: A Systematic Review

**DOI:** 10.7759/cureus.65769

**Published:** 2024-07-30

**Authors:** Bosten A Loveless, Kayvon A Moin, Phillip C Hoopes, Majid Moshirfar

**Affiliations:** 1 Ophthalmology, Hoopes Vision Research Center, Draper, USA; 2 Ophthalmology, Rocky Vista University College of Osteopathic Medicine, Ivins, USA; 3 Ophthalmology, American University of the Caribbean School of Medicine, Cupecoy, SXM; 4 Ophthalmology, Hoopes Vision, Draper, USA; 5 John A. Moran Eye Center, University of Utah School of Medicine, Salt Lake City, USA; 6 Eye Banking and Corneal Transplantation, Utah Lions Eye Bank, Murray, USA

**Keywords:** corneal collagen cross-linking (cxl), smile, prk, lasik, optical coherence elastography, corneal resistance factor, corneal hysteresis, young's modulus, corneal biomechanics, ocular response analyzer

## Abstract

Corneal biomechanical data has been used since 2005 to screen for keratoconus and corneal ectasia by corneal specialists. Older technology uses force applanation techniques over a 3 mm area in the central cornea, making it highly dependent on extraneous variables and unable to calculate the elasticity of the tissue. Brillouin microscopy is a newer method that uses a natural shift in the frequency of light as it passes through a material. This frequency shift can be used to estimate the viscoelasticity of the tissue. The advantage of Brillouin microscopy is that it can create a full three-dimensional (3D) map of the entire cornea without direct contact. A literature search was conducted using the databases PubMed, Google Scholar, and Ovid regarding the applications of Brillouin microscopy in corneal diagnostics. A final total of 16 articles was included describing the various ex vivo and in vivo studies conducted using Brillouin microscopy. Applications of this technology spanned from keratoconus diagnosis to post-corneal refractive surgery evaluation. All studies evaluated corneal biomechanics and other corneal properties through the quantification of Brillouin frequency shifts. Many of the studies found that this diagnostic device is capable of detecting subtle changes in corneal thickness and biomechanics in keratoconic corneas at a high level of specificity and sensitivity. However, limitations of Brillouin microscopy may include the duration of time required for use and fluctuations in accuracy depending on the corneal hydration state. Future technology seems to be geared toward a combination of optical coherence tomography (OCT) and Brillouin microscopy, using OCT as a three-dimensional pupil-tracking modality. Further research and understanding of the technology involved will lead to better care of patients in the field of ophthalmology.

## Introduction and background

The cornea is a crucial component of the eye's total optical power, serving as the primary refractive surface along with the tear film. Minor abnormalities or alterations in its shape can have profound effects on optic image formation [1]. Glycosaminoglycans (GAGs) and proteoglycans (PGs) in the extracellular matrix (ECM) as well as stromal collagen lamellae provide the cornea with viscoelastic qualities. Thus, the cornea is able to absorb and dissipate energy as heat as it deforms in response to an external load [2]. Several corneal biomechanical properties have been defined to better quantify the cornea's viscoelastic characteristics and its implications in predictive surgical outcomes and ocular diseases such as keratoconus and ectasia. Corneal hysteresis (CH), a property describing the viscous damping ability of the cornea, and corneal resistance factor (CRF), an indicator of the overall resistance of the cornea, have been measured and utilized as the main parameters of corneal biomechanics [3].

Various non-destructive in vivo methods can be used to measure corneal biomechanics, but the Ocular Response Analyzer (ORA) (Reichert, Depew, NY) is the most commonly used tool in clinical practice [3]. Using a puff of air to deflect the cornea, the ORA uses an infrared beam to track changes in the anterior cornea during inward and outward deviation, allowing the quantification of CH and CRF [4]. CH is measured as the difference in air pressures between force-in applanation (P1) and force-out applanation (P2) or (P1 - P2). CRF is measured as (P1 - kP2), where k is a constant derived from empirical analysis of the relationship between P1 and P2 and central corneal thickness (CCT) [3,4]. Despite the beneficial utility of the ORA, several limitations have been identified with this modality. In general, the clinical usefulness of the ORA can be called into question due to the nature of the values it measures. CH is a dynamic value that changes after intraocular pressure-lowering interventions are implemented [5]. CH also has a relatively low sensitivity value of 75% [6]. The ORA itself is very uncomfortable for many patients because of the air puff that it utilizes. Additionally, any decentralized irregularities of the cornea may not be detected by the ORA because it measures a 3-4 mm central area of the cornea. Central corneal surface irregularities can cause light scattering, which can distort the specular waveform, causing the machine to confuse tissue response for a surface response [7].

Corvis ST (Oculus, Wetzlar, Germany) is another one-dimensional method of assessing corneal biomechanics similar to the ORA, also relying on air puff tonometry. It provides a higher level of detail about corneal response by using high-speed Scheimpflug imaging [8]. It provides a value called Corneal Biomechanical Index (CBI) that has been shown to improve keratoconus and corneal ectasia detectability [9,10]. However, like ORA, it fails to measure corneal elasticity and also lacks the ability to three-dimensionally map the cornea [8]. Other methods have been devised to accomplish this feat, such as ultrasound surface wave elastometry (USWE), optical coherence elastography (OCE), and magnetic resonance elastography (MRE). USWE and MRE have been used in clinical applications such as cancer diagnosis but are limited in their application to corneal surface mapping due to a lack of appropriate resolution and difficulty determining boundaries of corneal lesions [11]. OCE overcomes the resolution issue that USWE and MRE have by providing sub-nanometer resolution through phase-sensitive techniques. However, depending on the type of OCE used, high levels of computational analysis and modeling are required [11]. Wave-based OCE requires a little eye movement and a standardized stimulation approach to prevent varying mechanical wave velocities. Natural frequency OCE is a very promising technology but requires many more studies to decorrelate the relationship between corneal frequencies and surrounding ocular parameters [11]. Applanation OCE is similar to ORA and Corvis ST in the fact that it uses air puff tonometry. Unfortunately, because of this, it also suffers similar limitations [12].

**Figure 1:**
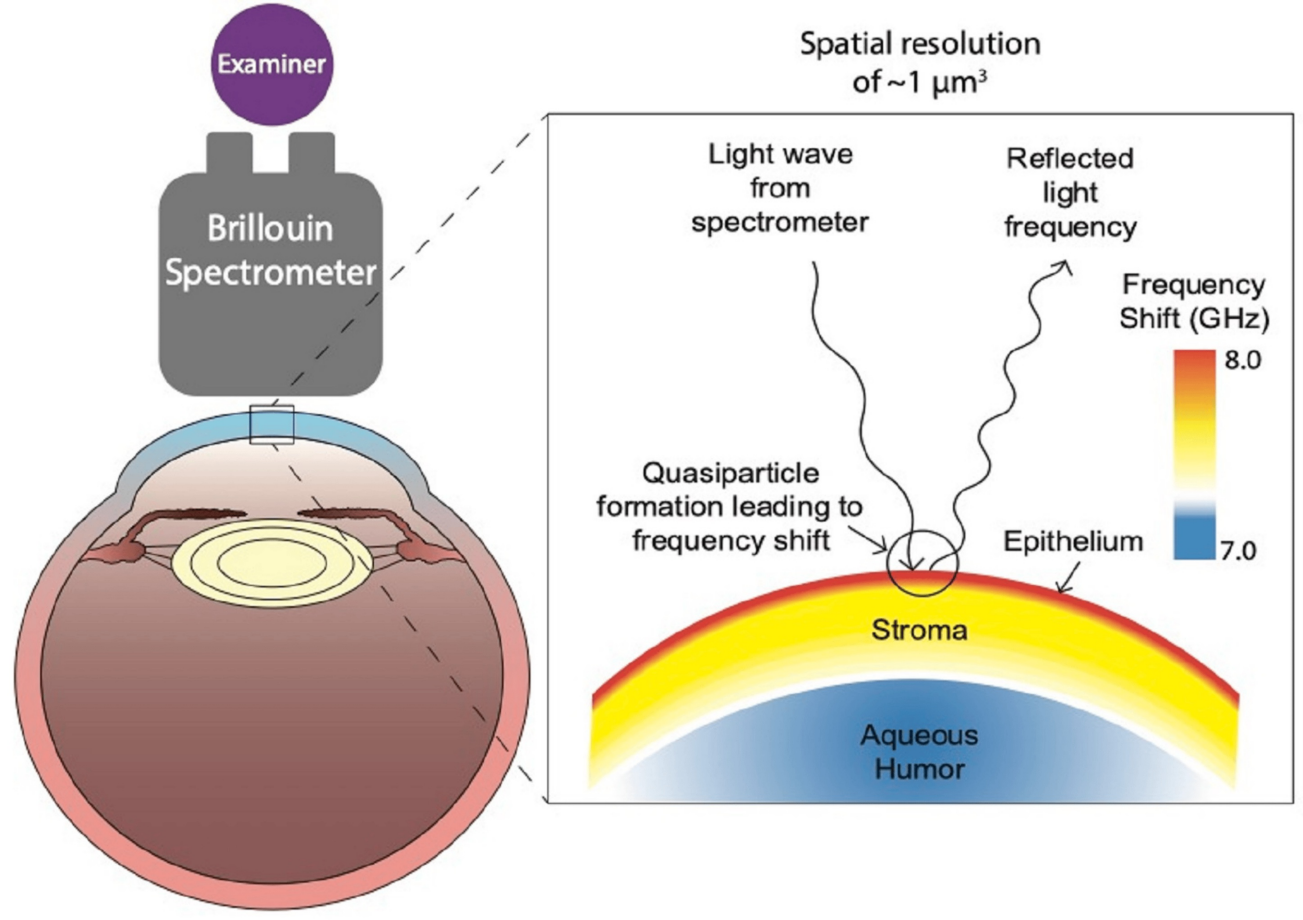
Diagram illustrating the mechanisms of Brillouin microscopy Created with Adobe Illustrator by the authors

French physicist Léon Brillouin first discovered the concept of light waves interacting with the natural waves within a material in 1922 [13]. This process is modulated by the refractive index of the material. The result of this interaction is that a portion of the light wave changes in frequency or energy level. This occurs through either photon energy loss or gain through a process called quasiparticle formation or absorption [13]. Brillouin microscopy was created using this frequency shift. Figure 1 further illustrates the process to acquire such data. The patient is seated across from an examiner, and a near-infrared light is shone into the patient's eye, and the reflected frequency shift is recorded by the spectrometer. The frequency shift can be used to estimate the viscoelasticity of a material by calculation of the longitudinal viscoelastic modulus (LVM) [14]. It should be noted that the LVM is not equal to the elasticity or stiffness that a person would feel by directly pressing on the cornea. This is better represented by Young's modulus, which represents a proportion of the stress and strain caused by deforming a material [15]. This value is found in elastography testing methods such as USWE, MRE, and OCE. However, the LVM will increase or decrease in the same direction as Young's modulus as pathological changes occur [8]. There is currently no known method to convert between LVM and Young's modulus [16]. The advantage of using the LVM is that it is more standardized than Young's modulus, which can vary from device to device due to the lack of a standardized stress-strain region [17,18].

Our objective with this review is to synthesize the current literature regarding the clinical applications of Brillouin microscopy for corneal diagnostics. We aim to highlight its advantages, limitations, and future directions for adoption into clinical practice.

## Review

**Figure 2:**
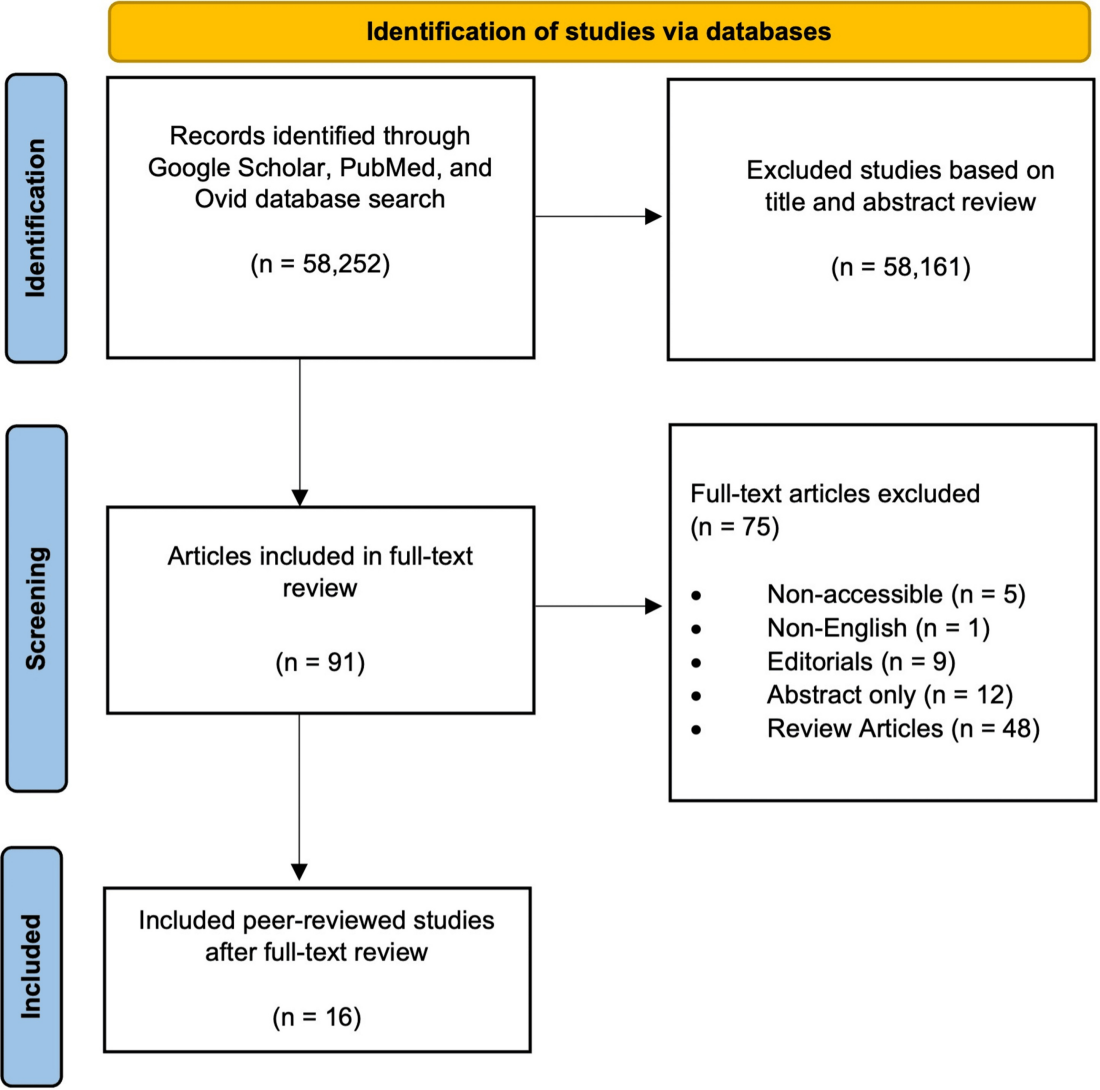
Flow diagram process of the literature search regarding the applications of Brillouin microscopy in corneal diagnostics Created from the diagram template from www.prisma-statement.org by the authors

A literature search regarding the applications of Brillouin microscopy was conducted through June 10, 2024, with Google Scholar, PubMed, and Ovid search engines using the following terms: "astigmatic keratotomy," "conductive keratoplasty," "cornea," "corneal refractive surgery," "femtosecond lenticule extraction," "intracorneal ring," "laser-assisted in situ keratomileusis," "LASIK," "laser-assisted subepithelial keratomileusis," "LASEK," "laser thermal keratoplasty," "photorefractive keratectomy," "PRK," "radial keratotomy," "refractive," "refractive surgery," "small incision lenticule extraction," "SMILE," "crosslinking," "CXL," and "keratoconus." The format for these search terms was created using the Boolean operator "AND" to combine each of these terms with the phrase "Brillouin microscopy." This yielded an initial total of 58,252 articles (Figure 2). After thorough title/abstract screening, 58,161 articles were excluded due to irrelevance. The remaining 91 articles were full-text reviewed and screened. Non-accessible (five), non-English (one), editorials (nine), abstracts (12), and reviews (48) were excluded. This resulted in a final total of 16 articles. These articles were organized by "ex vivo" (10) or "in vivo" (seven) and tabulated.

Table 1 summarizes the 10 laboratory ex vivo studies found through the literature search that utilized Brillouin microscopy [19-28]. The sample sizes of these studies ranged from four to 44 eyes and consisted of bovine, leporine, porcine, or human eyes. Procedures that were utilized in the studies included corneal cross-linking (CXL) or laser-assisted in situ keratomileusis (LASIK) flap creation with subsequent CXL. All studies used Brillouin microscopy to evaluate corneal biomechanics in different scenarios. For example, Scarcelli et al. [21] measured Brillouin frequency shifts in keratoconic and normal human corneas and found that these shifts were significantly lower in the anterior portion of keratoconic corneas compared to healthy corneas (7.99 GHz versus 8.17 GHz, p<0.001). They additionally demonstrated that Brillouin microscopy was sensitive enough to detect decreases in shifts at the apex of the keratoconic cone compared to regions away from the cone. Randleman et al. [23] studied porcine corneas and evaluated biomechanical changes after LASIK flap creation and with subsequent rapid CXL with Brillouin microscopy. They found that there was a significant reduction in Brillouin frequency shifts after flap creation across the total corneal thickness (-0.035 GHz, p=0.0195) and anterior stromal region (-0.104 GHz, p=0.0039) as well as an insignificant increase in shifts after rapid CXL.

**Table 1:** Ex vivo studies utilizing Brillouin microscopy CXL = corneal cross-linking, LASIK = laser-assisted in situ keratomileusis, RB = rose bengal, L-CXL = localized corneal cross-linking, CACXL = contact lens-assisted CXL, S-CXL = standard CXL

Author	Study design	Eyes	Procedure(s)	Purpose	Results
Scarcelli et al. [19] (2011)	Laboratory	4 (bovine)	CXL	The feasibility of Brillouin microscopy as a method to test the biomechanics of the cornea	Increase in Brillouin shifts after CXL from 7.5 to 8.2 GHz frequency prior to procedure, indicating an increase in biomechanical stability
Cherfan et al. [20] (2013)	Laboratory	44 (leporine)	CXL	To assess corneal stiffening with Brillouin microscopy after RB plus green light administration for keratoconus	Increase in Brillouin shifts/scattering in the anterior cornea (~100 micron) indicating increased stiffness
Scarcelli et al. [21] (2014)	Laboratory	18 (human)	-	To evaluate if Brillouin microscopy can differentiate the mechanical properties of keratoconic corneas versus healthy corneas	Mean Brillouin shift in anterior keratoconic cornea lower than healthy corneas (7.99 versus 8.17 GHz; p<0.001); shifts in regions away from the apex of cone higher than within cone region
Lepert et al. [22] (2016)	Laboratory	5 (bovine)	CXL	To assess corneal biomechanics of corneal tissue with Brillouin microscopy	Anterior cornea in CXL-treated corneas showed significantly higher Brillouin shifts compared to untreated tissue (~8.0 GHz versus 6.5 GHz), indicating stiffer regions
Randleman et al. [23] (2017)	Laboratory	11 (porcine)	LASIK flap creation CXL	To evaluate biomechanical changes after LASIK flap creation and rapid CXL measured with Brillouin microscopy	Reduction in Brillouin shift after flap creation across total corneal thickness (-0.035 GHz, p=0.0195) and anterior stromal region (-0.104 GHz, p=0.0039); a small increase in shift after rapid CXL (p>0.05)
Webb et al. [24] (2019)	Laboratory	10 (porcine)	CXL	To investigate the stiffening effect of L-CXL beyond irradiated regions with Brillouin microscopy	Broad transition zone of 610 microns in width observed between fully cross-linked and non-cross-linked sections of the cornea
Zhang et al. [25] (2019)	Laboratory	30 (porcine)	CXL	To evaluate the impact of CACXL and S-CXL on corneal stiffness with Brillouin microscopy	Anterior corneal stiffening more pronounced in CXL compared to CACXL (longitudinal modulus: 7.8% versus 5.5%, p<0.001)
Webb et al. [26] (2020)	Laboratory	41 (Human)	CXL	To detect the mechanical anisotropy of the cornea using Brillouin microscopy	Brillouin shift increases of 53 MHz and 96 MHz when probed from 0 to 15 and 30 degrees along the direction of stromal fibers; Brillouin microscopy did not lose sensitivity up to 96% water content
Zhang et al. [27] (2020)	Laboratory	24 (Porcine)	LASIK flap creation CXL	To compare corneal biomechanics after CXL over S-CXL or under the LASIK flap (flap-CXL) using Brillouin microscopy	Higher stiffening impact with S-CXL in the anterior corneal stroma (8.40 GHz versus 8.22 GHz, p<0.001); minimal stiffening in the middle or posterior cornea with either protocol
Eltony et al. [28] (2022)	Randomized controlled trial	8 (porcine/human)	-	To quantify anisotropic mechanical properties and longitudinal moduli with Brillouin microscopy	It is possible to derive a mathematical model of the cornea using the calculated longitudinal moduli, allowing for detailed simulation of corneal diseases

Table 2 summarizes the six in vivo studies found through the literature search describing the applications of Brillouin microscopy [29-34]. The study designs included retrospective case-control studies, case studies, and prospective comparative, case-control, and cross-sectional studies. The sample sizes of these studies ranged from one to 93 eyes. All studies assessed corneal biomechanics with Brillouin microscopy either after corneal procedures such as small incision lenticule extraction (SMILE), laser-assisted subepithelial keratomileusis (LASEK), photorefractive keratectomy (PRK), CXL, or in virgin corneas. For example, Seiler et al. [31] demonstrated a significant correlation between age and central Brillouin shift, indicating corneal stiffening with each year of increasing age. However, when examining keratoconic corneas, they concluded that Brillouin microscopy was neither specific nor sensitive for the precise differentiation of the severity stages of keratoconus. With regard to corneal procedures, Zhang et al. [33] showed that Brillouin microscopy modified with three-dimensional (3D) motion-tracking was able to identify biomechanical changes after LASIK, PRK, and CXL. They found focal corneal biomechanical differences between normal eyes, eyes with stage I and II keratoconus, and eyes that had remotely undergone successful laser vision correction by assessing Brillouin frequency shift values. Shao et al. [30] and Randleman et al. [34] found that when compared to standard Scheimpflug methods for assessing corneal biomechanics, Brillouin microscopy was more sensitive in detecting subtle differences between keratoconic and normal corneas.

**Table 2:** In vivo studies utilizing Brillouin microscopy PRK = photorefractive keratectomy, LASIK = laser-assisted in situ keratomileusis, CXL = corneal cross-linking, OCT = optical coherence tomography, LVC = laser vision correction

Author	Study design	Eyes	Procedure(s)	Purpose	Results
Scarcelli et al. [29] (2012)	Case study	1	-	To report the first Brillouin measurement of a human eye in vivo	5.25-5.5 GHz Brillouin shifts corresponding to the thickness of the cornea
Shao et al. [30] (2018)	Prospective comparative study	93	-	To demonstrate the diagnostic potential of Brillouin microscopy in the screening and diagnosis of keratoconus	Determined cone location of different keratoconic corneas at different severity stages using Brillouin shifts; assessed differences in shifts between normal and keratoconic corneas
Seiler et al. [31] ( 2019)	Retrospective case-control study	83	-	To investigate the correlation between age and Brillouin frequency shift and compare normal corneas to keratoconic ones	Significant correlation between age and central Brillouin shift: increase in shift of 4 MHz per decade in normal cornea (p=0.011); Brillouin microscopy neither specific/sensitive for precise differentiation of keratoconus stages
Zhang et al. [32] (2022)	Case study	1	-	To use 3D motion-tracking Brillouin microscope for in vivo corneal biomechanics mapping	Demonstrated that by adding 3D pupil tracking and combining Brillouin technology with OCT, measurements can get the appropriate sensitivity needed to do in vivo measurements clinically
Zhang et al. [33] (2023)	Prospective cross-sectional study	30	LASIK, PRK, CXL	To compare biomechanical differences between normal, keratoconic, and post-LVC corneas using motion-tracking Brillouin microscopy	Motion-tracking Brillouin microscopy identified focal corneal changes after refractive surgery, performing better than or equal to existing clinical methods
Randleman et al. [34] (2024)	Prospective cross-sectional study	30	-	To characterize the focal biomechanical alterations that occur in keratoconic eyes using motion-tracking Brillouin microscopy and evaluate its ability to differentiate normal eyes from keratoconic ones	Significant differences in Brillouin shifts between keratoconic and normal corneas in mean and minimum plateau, and mean and minimum anterior 150 micron metrics (p<0.001); Brillouin microscopy outperformed two-dimensional methods of screening

Due to the novelty of Brillouin microscopy, there were relatively few studies involving refractive surgery specifically. However, the direction this technology seems to be taking is toward a combined method using OCT to help with motion tracking as well as Brillouin techniques to create a full three-dimensional map of the cornea [35]. With this adjustment, studies have shown that a specificity and sensitivity of 100% may be obtained [34]. For reference, the sensitivity of CH is 87% with a specificity of 65%, while CRF shows a sensitivity of 90.5% and specificity of 66% when measured with the ORA [36]. This may be attributed to the fact that ORA and Corvis ST rely on an applanator that only covers a diameter of 3 mm in the central cornea, whereas Brillouin microscopy coupled with motion-tracking technology is able to create a full three-dimensional map and have precise pupil fixation during the examination [33]. Randleman et al. [34] demonstrated, through their use of motion-tracking Brillouin microscopy, that they found significant differences in Brillouin shifts between normal and subclinical keratoconic corneas. This identification suggests parameters or values for subclinical keratoconus cutoffs, highlighting motion-tracking Brillouin microscopy as an efficacious screening tool.

Despite its applications and clinical advantages, Brillouin microscopy has certain limitations. Unfortunately, it is difficult to incorporate into clinical practice due to total data acquisition time. It typically takes four to eight minutes to fully sample the tissue, and any motion or blinking during that time could cause artifacts and inaccurate data [30,35,37]. Zhang et al. [32] additionally reported that a complete scan took approximately 20 minutes to perform accurately. Conversely, Scheimpflug diagnostic tools such as the ORA or Corvis ST take approximately one to three minutes to perform, making these methods more efficient and streamlined accessories to clinical practice. Another limitation is the potential fluctuations in Brillouin measurements due to external factors. Shao et al. [37] demonstrated that Brillouin frequency shifts varied by up to 10 MHz due to physiological changes in corneal hydration levels. Acknowledging that there may be diurnal fluctuations in physiological corneal hydration [37], this could produce unpredictable and unreliable results for patients, thereby confounding the utility of Brillouin microscopy.

## Conclusions

Based on the comprehensive review of Brillouin microscopy and its applications in assessing corneal biomechanics, it is evident that this technology holds significant promise in clinical ophthalmology. By leveraging the principles of light wave interaction with materials, Brillouin microscopy provides a unique insight into the viscoelastic properties of the cornea, which are crucial for understanding conditions such as keratoconus and evaluating outcomes of refractive surgeries. However, despite its advantages, Brillouin microscopy faces practical challenges that hinder its widespread clinical adoption such as duration of data acquisition time and sensitivity of measurements to physiological changes in corneal hydration.

Moving forward, addressing these technical challenges could enhance the feasibility and integration of Brillouin microscopy into routine clinical practice. Advances in motion-tracking technology and optimization of scan protocols may reduce acquisition times and mitigate the impact of patient-related artifacts. Furthermore, future studies focusing on refining the correlation between Brillouin measurements and clinical outcomes in larger patient cohorts are warranted to establish its utility in guiding clinicians for appropriate diagnosis and management.
